# Bilateral Corpus Cavernosum and Complete Urethral Rupture: A Double Trouble

**DOI:** 10.7759/cureus.15481

**Published:** 2021-06-06

**Authors:** Doğukan Sökmen, Yusuf İ Çömez

**Affiliations:** 1 Urology, Memorial Bahçelievler Hospital, Istanbul, TUR; 2 Urology, Üsküdar University, Istanbul, TUR

**Keywords:** penile fracture, urethral rupture, corpus cavernosum

## Abstract

Penile fracture is a rare urologic emergency. The main finding is a partial disruption of one or both cavernosal bodies due to blunt trauma of the penis during an erection. Complete or partial injury of the urethra may accompany the penile fracture but complete urethral rupture is rarely encountered. In this study, we present the management of a penile fracture case with disruption of both corpus cavernosum with total urethral rupture.

## Introduction

Penile fracture (PF) is defined as the rupture of the tunica albuginea and corpora cavernosa, often caused by blunt trauma to the penis during sexual intercourse [1]. The coexistence of PF and urethral injury is not a common condition, and it has been reported in the range of 8-23% [2]. Besides PF symptoms like penile pain, a popping or cracking sound and rapid loss of erection, blood in the urethral meatus, hematuria, and urinary retention should be assessed as a sign of urethral injury [3]. Urgent surgical repair is recommended to prevent complications such as erectile dysfunction, penile curvature, and urethral stricture [4].

This study aimed to present a case with bilateral corpus cavernosum and complete urethral rupture after blunt trauma to the penis during sexual intercourse and describe the surgical technique applied.

## Case presentation

A 21-year-old male patient presented to our emergency clinic two hours after blunt injury of the penis during sexual intercourse. He expressed that he had been forced towards the perineum of his partner at the beginning of the intercourse. He heard a cracking sound and afterward had severe pain, detumescence, and little blood at the urethral meatus. He had not yet tried to void. Tumefaction and hematoma over the entire penile shaft were seen during physical examination. There was a palpable defect at the ventral shaft of the penis. The scrotal examination was normal. The bladder was not distended. Ultrasonography (USG) showed hematoma and irregularity of corpora cavernosum at the mid-ventral side of the penis. The patient was informed about the potential postoperative complications and the risks of choosing a conservative approach. He underwent emergency surgery after his approval.

**Figure 1:**
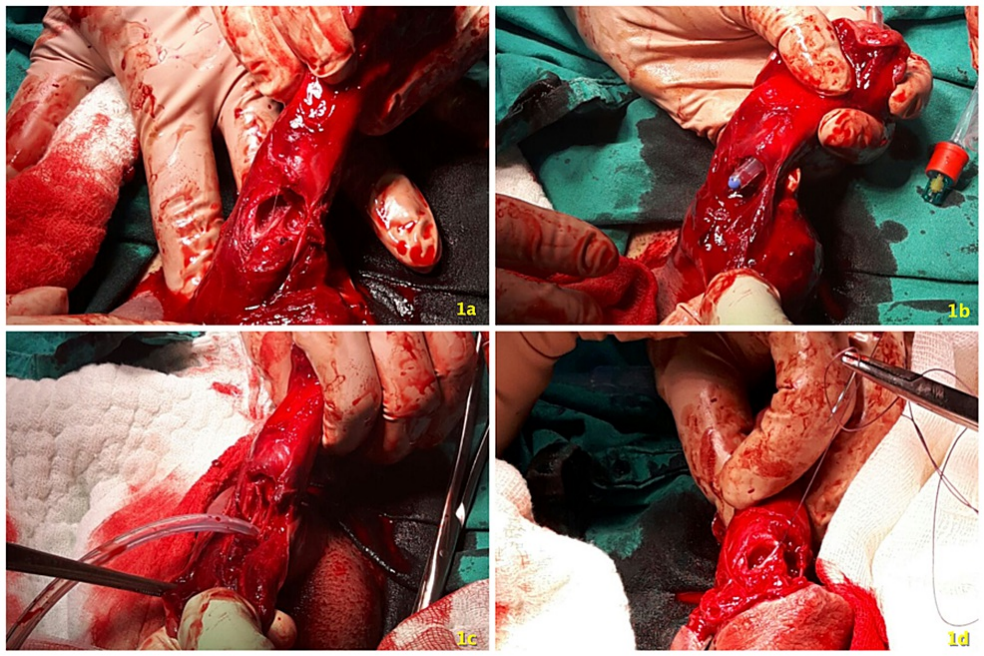
Bilateral cavernosal rupture (a). Identifying urethra with an 18-French catheter (b). Checking proximal urethra (c). Suturing cavernosal defects separately (d).

After fluid infusion and antibiotic prophylaxis, under spinal anesthesia, a penile subcoronal circumferential incision was performed and the hematoma was evacuated. Both cavernosal ruptures on the mid-ventral side of the penis were clearly seen but the urethra could not be identified at the level of the defect (Figure 1a). An 18-French urethral catheter was inserted from the meatus towards the disrupted urethra (Figure 1b). The integrity of the proximal part of the urethra was checked via insertion of the catheter into the bladder (Figure 1c). Corpus cavernosal defects were repaired separately by absorbable polydioxanone stitches (Figure 1d).

**Figure 2:**
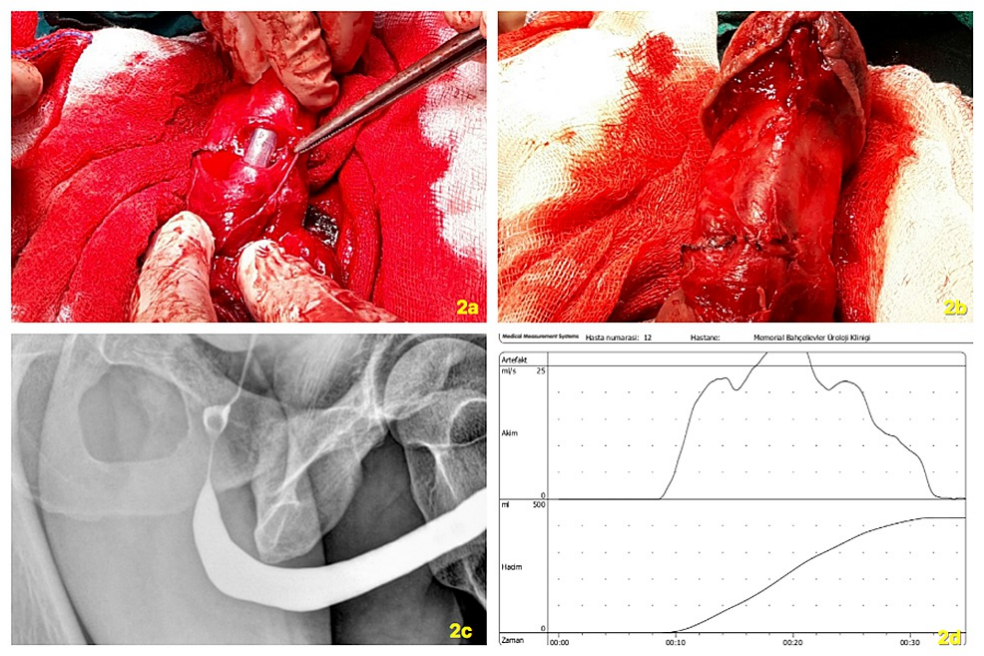
Urethral reconstruction (a). Both cavernosal and urethral defects are repaired (b). Retrograde urethrography at postoperative third month (c). Uroflowmetry at postoperative third month (d).

The end-to-end urethral anastomosis was performed with 4/0 Vicryl separate stitches with the guidance of the urethral catheter (Figures 2a, 2b). 

The patient was discharged two days after surgery. The urethral catheter was removed on the 14th postoperative day and he could void normally. Diazepam has been prescribed for painful erection at the early postoperative period. He had normal erectile and voiding functions one month later. A point defect was palpable on the ventral side of the penis shaft at physical examination and he expressed a slight (5-10°) penile curvature on the left side during erection without difficulties during sexual intercourse. At a three-month follow-up, the patient was evaluated with retrograde urethrography (RGU) that documented a regular urethral profile without signs of stricture neither fistula (Figure 2c). Uroflowmetry demonstrating an average flow rate of 16.6 ml/s and a maximum flow rate of 30.6 ml/s (Figure 2d). Validated questionnaires demonstrated optimal functional outcomes. (International Prostate Symptoms Score (IPSS) =3 and International Index of Erectile Function-5 (IIEF-5) = 23) (Figure 2d).

## Discussion

Penile fractures are rare urologic emergencies with an incidence of one in 175,000 cases [5]. Bilateral corpus cavernosal rupture is a rare event and is observed in only 10% of the PF cases. The history and clinical examination are usually adequate for the diagnosis [6]. In case of suspicion in clinical diagnosis, USG, cavernosography, and MRI may be helpful [7]. Surgery and a conservative approach are the treatment modalities for penile fracture. Immediate surgical repair is the most chosen treatment modality and provides better outcomes and fewer complications than conservative management [8]. Postoperative complications, such as erectile dysfunction, penile plaques and nodules, chordee, and penile pain, are the main complications [4]. Conservative treatment may lead to an increased risk of complications [7]. In our case, early surgical intervention was chosen according to USG, clinical signs, and physical examination.

Associated urethral injury rates vary from 8% to 23% [2]. A concomitant urethral injury should always be suspected if the presence of blood at meatus, microscopic or gross hematuria, and disorders of voiding are encountered [4]. However, these symptoms are not always present in all patients with urethral injury. In a systematic review published recently, it was stated that 50% of urethral injuries are asymptomatic and detected incidentally by USG or intraoperatively [3]. However, RGU has a false negativity rate of 28.5% [9]. Additionally, Kamdar et al. recommended the simultaneous use of flexible cystoscope for PF cases with suspected urethral injury [10]. Although the sensitivity of the MRI examination for penile fracture is reported as 100% and the specificity is 77.8%, its sensitivity for concomitant urethral trauma has been reported as 60% and specificity as 78.3% [11]. In our case, we did not perform MRI as suspicion of penile fracture was high from clinical examination/ultrasound.

Urethral injuries should be treated according to the size of the injury. For partial injuries, urinary diversion or primary suturation of the rupture can be adequate [4]. However, for complete urethral transections, complex procedures such as tension-free anastomotic or augmentation urethroplasty are needed [2].

Postoperative urethral catheterization time should be determined according to the complexity of the injury. Catheterization is required for 10-14 days in the presence of partial injury and 14-21 days in the presence of complete rupture [12]. The baseline diagnostic methods recommended to evaluate postoperative voiding functions are the IPSS questionnaire and uroflowmetry [3]. In some studies, 30% of the cases with PF who underwent urethral reconstruction have been found to be impaired in IPSS [12,13]. Urethral stricture, pseudoaneurysm, urethrocutaneous fistulas, and related subcutaneous abscesses may develop in the postoperative long term [4]. In our case, the patient did not have any early or late complications. He had a regular sexual relationship with no anxiety.

## Conclusions

Penile fracture is a rare urological emergency. The main problems are late diagnosis and surgical intervention. Although partial urethral rupture may accompany the penile fracture occasionally complete urethral rupture is very rare. In case of penile fracture, the concomitant urethral rupture has to be suspected. Immediate surgical repair will ensure satisfactory results and lesser complications.
